# Effectiveness of animal-assisted services for school-aged children: a systematic review

**DOI:** 10.1007/s00787-025-02740-7

**Published:** 2025-06-02

**Authors:** Ingyin Moe, Pei Ju Ho, Maria Andersson, Sara Karlberg, Lena Lidfors, Filipa Sampaio, Inna Feldman

**Affiliations:** 1 Department of Public Health and Caring Sciences, Uppsala University, Uppsala, Sweden; 2 Department of Applied Animal Science and Welfare, Swedish University of Agricultural Sciences, Uppsala, Sweden; 3 Svenska Terapihundskolan (Swedish schools for therapydogs), Strömsholm, Sweden; 4 Department of Epidemiology and Global Health, Umeå University, Umeå, Sweden

**Keywords:** Animal-assisted services, School-going children, Behavior, Well-being, Mental health, Systematic review

## Abstract

Animal-assisted services (AAS) have demonstrated potential benefits for children and adolescents, particularly in emotional development and educational outcomes. However, existing systematic reviews often focused on specific populations or types of animals, and overlook the quality of included studies. This review evaluates the effectiveness of animal-assisted services on the well-being and behavior of school-aged children, and assesses the risk of bias of the included studies. Following PRISMA Guidelines, a systematic search was conducted in MEDLINE, CINAHL, PsycARTICLES, PsycINFO via EBSCOhost, Cochrane Database of Systematic Reviews, and Web of Science, from database inception until August 15, 2023. Risk of bias was assessed using the Cochrane Risk of Bias 2 for randomized trials and ROBINS-I for non-randomized studies. Two reviewers (IM, PJH) assessed each record, and two additional reviewers (IF, FS) resolved disagreements. Data were synthesized narratively, and presented in tables and figures. From 2,380 initial articles, 30 met the inclusion criteria. Studies were primarily randomized controlled trials (*n* = 21), involving various populations, from children with autism spectrum disorders (*n *= 10) to general population (*n* = 8). Outcomes were grouped into abilities, attitudes, externalizing behaviors, internalizing behaviors, disorder-specific measures, and well-being. AAS showed positive impacts on socio-emotional functioning and behavior despite diverse intervention approaches. Nonetheless, all studies had study design problems, which caused high risk of bias. AAS appears to enhance cognitive, behavioral, and socio-emotional aspects, as well as learning in school-aged children. Further methodologically rigorous research and evidence, such as implementation studies and cost-effectiveness analyses comparing AAS to standard practices are needed to support decision-making.

**Supplementary Information:**

The online version contains supplementary material available at 10.1007/s00787-025-02740-7.

## Introduction

In recent years, incorporating animals into educational and therapeutic settings for children and adolescents has advanced significantly, especially in the United States [1]. Various concepts are used to describe this practice, such as animal-assisted interventions, activities, or therapy [2, 3]. Due to lack of consistent terminology, Binder et al. [4] have proposed an umbrella term called animal-assisted services (AAS) which encompasses three categories of interventions: animal-assisted treatment (AATx), animal-assisted education (AAE), and animal-assisted support programs (AASP) [4]. According to Binder et al. [4], AAS involves specially trained animals under professional guidance to enhance human well-being through therapeutic, educational, or supportive processes. The administration of AAS varies by animal type (e.g., dogs, dolphins, horses), setting (e.g., schools, hospitals, stables), duration, and whether delivered in groups or individually [5].

AAS positively impacts the social, emotional, cognitive, and physical development of children and adolescents, as shown in both theoretical frameworks and several scientific research [2, 6–8]. A meta-analysis conducted on various types of AAS noted positive findings in medical well-being and behavioral outcomes, especially when AAS are incorporated into established interventions [5]. In the context of education, there is ample evidence on animal presence enhancing learning for school-aged children [9–15]. A systematic review by Brelsford et al. [16] found direct and indirect positive effects on education, particularly in socio-emotional areas like emotional regulation, mood, and social behaviors [16]. Further supporting this notion that AAS can impact learning, Gee et al. [17] presented a theoretical framework based on existing research [17]. The framework outlines how learning is indirectly influenced through motivation, engagement, self-regulation, and human social interaction. AAS is therefore considered to positively enhance the learning environment through its impact on motivation and engagement as well as social skills.

Moreover, AAS has been shown to benefit various conditions and disorders. Meta-analyses and reviews by O’Haire [18], Mapes and Rosén [19], and Dimolareva and Dunn [20] found that AAS positively impacts children with autism spectrum disorders (ASD), improving social interaction, communication, problem behaviors, autistic severity, and stress [18–20]. Additionally, AAS has demonstrated several positive effects for children with various challenges, such as increased school attendance and reduced hospitalizations in children with acute mental disorders [21], enhanced attachment security in children with attachment problems [22], reduction in symptoms severity in those with attention deficit hyperactivity disorders (ADHD) [23, 24] and Down syndrome [25].

Several systematic reviews have attempted to synthesize the empirical evidence on AAS for children and adolescents. However, these reviews often focused on children with specific conditions, certain type of study designs such as within-group studies, or limited settings such as hospitals [18, 26–29]. Only one review summarized the evidence of AAS in educational contexts [16]. Most often, the authors did not perform a quality assessment of the studies included or meta-analysis, or included studies without control groups. The latter has been raised extensively in past studies as appropriate control groups are needed to establish that observed changes in outcomes are associated with the intervention and not a result of natural occurrences over time [30, 31]. Therefore, there is a need for further evidence synthesis and a clearer understanding of AAS benefits for school-aged children. To address this, we conducted a systematic review of the existing literature on effects of AAS, synthesized the evidence found, and assessed the risk of bias of the included studies.

## Methods

### Search strategy and selection criteria

This systematic review followed the 2020 Preferred Reporting Items for Systematic Reviews and Meta-Analysis (PRISMA) guideline [32], and was registered in PROSPERO (ID CRD42023453110). The electronic databases PubMed/Medline, PsycARTICLES, PsycINFO, and CINAHL via EBSCOHost, Cochrane Database of Systematic Reviews, and Web of Science were searched from inception up to August 15, 2023. The population, intervention, control, outcomes, and study design (i.e. PICOS) criteria were used to guide the search strategy detailed in Table 1 [33].

**Table 1:** PICOS and selection criteria used

PICOS Component	Inclusion Criteria	Exclusion Criteria
Population: School-aged children and adolescents.	School-aged children is defined as children aged 5–18.	No population restrictions.
Intervention: Animal-assisted interventions in non-healthcare settings.	Interventions with any animal e.g. horses in school, or school-associated facilities such as barns.	Interventions with robotic animals, or in healthcare settings such as hospitals.
Comparator: Any control or comparator without animals.	Active i.e. alternative intervention, or passive control e.g. waitlist.	Studies without a control group, or one that involved animals.
Outcomes: School-related, well-being, and/or effects on behavior.	Effects on behavior encompass internalizing and externalizing. School-related outcomes include children attitudes and abilities. Well-being is approached through health-related quality of life (HrQoL) measures.	Studies focused solely on executive function skills without broader behavioral or school-related outcomes, or clinical outcomes other than behavior/well-being measured in the school settings. Studies with insufficient outcome data.
Study Design: Studies with a comparator.	Randomized control trials and observational studies.	Systematic reviews, grey literature, case studies, case reports, and studies with a within-subject design.

The inclusion criteria were studies with animal-assisted interventions in non-healthcare settings; randomized control trials and observational studies; studies with control groups; school-aged children aged 5–18 years; peer-reviewed articles; published in English, Portuguese, Spanish, Chinese, Russian, Italian or French. Previous reviews and literature were used to identify relevant keywords [26, 34]. Exclusion criteria were studies with robotic animals; studies in healthcare settings such as hospitals; studies without a control group or control group with animals; grey literatures and non-peer-reviewed.

The search terms used were: (child* or kid* or “school age*” or youth or adolescen* or “young people”) AND (“animal assisted” or “dog assisted” or “equine assisted” or “pet assisted” or “canine assisted” or “hippotherap*” or “pet therap*” or “animal therap*” or"assistan* dog*"or"horseback riding"or"pet facilitated"or"therapeutic animal*"or"therapeutic horse*"or"therapy with animal*"or"dolphin assisted") AND ("school performance"or"performance"or"school achievement*"or"school outcome*"or"academic achievement*"or"academic performance"or"learning"or"behavio* change"or"behavio*"or"school attend*"or “presen*” or “absent*” or “grade*” or “literac*” or “social skill*” or"quality of life"or “read*” or “class attend*” or “school absen*” or “class performance” or “cognitive skill*” or “motivation” or “anxiet*” or “student engag*” or “mental health” or “well being”). Using Rayyan, a web-based tool, two blinded reviewers (IM and PJH) manually removed duplicates and independently screened study titles and abstracts based on predefined criteria [35]. Disagreements were discussed until consensus was reached or referred to an additional reviewer (IF or FS). The reviewers'agreement indicated strong consensus, with only minor discrepancies observed during the screening of titles and abstracts (10%). Relevant articles were retrieved, and an open web search was conducted for missing full texts.

### Data extraction

One reviewer (IM) systematically extracted data from all included studies using a standardized Excel template, while a second reviewer (PJH) randomly extracted data from 16 (52%) of the studies, achieving a 90% consensus. Data extracted were: First Author, Year, Study Population Characteristics, Sample Size, Intervention Setting, Country, Intervention Characteristics, Type of animal, Time Points, Comparator Characteristics, Study Outcomes, Measurement Instruments, Study Design, and Effect Sizes. Mean and standard deviations of the effect sizes were extracted for all relevant outcomes at baseline, post-intervention, and any follow-up timepoints. Extraction details are available in Table 2. Authors of some included studies (*n* = 3) were contacted via email for missing outcome information.

**Table 2:** Types of data extracted from included studies

Types of Data	Information
First Author, Year	The year the article was published.
Study Population Characteristics	Age, sex, and any other specifics detailed in the study, such as children with ADHD.
Sample Size	Intervention and comparator sample sizes included in the study analyses.
Intervention Setting	Facility in which the intervention is implemented; retrieved directly from the study.
Country	Based on the intervention setting.
Intervention Characteristics	Main components and details, such as duration and frequency; retrieved directly from the study.
Type of animal	The animal, such as dogs or horses incorporated in the intervention.
Time Points	Time points in which the outcome measurements were conducted.
Comparator Characteristics	Main components and details, such as duration and frequency; retrieved directly from the study.
Study Outcomes	Any school-related, behavioral, and/or HRQoL outcome reported in the study.
Measurement Instruments	The instruments used, such as SDQ and CBCL, to measure each specific outcome reported in the study.
Study Design	Pre-defined as randomized controlled trial or observational study.
Effect Sizes	Mean effect sizes at baseline, post-intervention, and any other follow-up time points reported in the study.

Abbreviations: ADHD, Attention Deficit/Hyperactivity Disorder; HRQoL, Health-related Quality of Life; SDQ, Strengths and Difficulties Questionnaire; CBCL, Child Behavior Checklist

### Risk of bias assessment

Two independent reviewers conducted the bias assessment: IM assessed all studies, and PJH randomly reviewed 18 (64%) of the studies. Discrepancies were resolved through discussion. Two Cochrane-recommended tools were employed to assess risk of bias of the included studies— Risk of Bias 2 (RoB 2) for RCTs, and the Risk of Bias in Non-Randomized Studies of Interventions (ROBINS-I) for non-RCTs [36, 37]. RoB 2 evaluates bias in randomization, deviations from intended interventions, missing data, outcome measurement, and selective reporting by rating each domain as low, some concerns, or high risk. ROBINS-I evaluates bias in confounding, selection of participants, classification of interventions, deviations from intended interventions, missing data, outcome measurement, and selective reporting. Each domain was graded as having no information, low, moderate, serious, or critical risk. Both tools produced summary risk of bias results, based on the individual domain risks.

### Data synthesis

Given the heterogeneity of studies and outcomes included, a meta-analysis was not feasible and the evidence was synthesized narratively. The included studies were classified by population characteristics, and outcomes were grouped into externalizing behavior, internalizing behavior, abilities, attitudes, well-being, and disorder specific, as shown in Table 3. The decision to group the outcomes was done after discussions with experts in child development and psychology. An additional outcome group named “Composite” was made to separate subscales from total instrument scores. Outcomes not fitting into these categories were listed as “Other”. A narrative synthesis was used to synthesize the evidence by main study characteristics, intervention characteristics and outcomes. Additionally, risk of bias assessment was described and presented through tables and graphs.

**Table 3:** Definitions of the outcome groups used to summarize results

Outcome Group	Definition
Abilities	The capacity or skill to do something that benefits the child, such as goal-directed behavior or leadership.
Attitudes	A state of mind, accompanied by feelings and emotions that causes the child to approach or avoid a situation or task [38].
Externalizing Behavior	Behaviors that are manifested outwardly and reflect the child negatively acting on the external environment such as hyperactivity or rule-breaking [39].
Internalizing Behavior	Behaviors that are directed inwardly and represent an overcontrolled and inner-directed pattern of behavior such as social withdrawal and anxiety [39].
Well-being	A positive state experienced by individuals and societies. This encompasses quality of life, as well as the ability to contribute to the world in accordance with a sense of meaning and purpose [40].
Disorder Specific	Measures from instruments pertaining a specific disorder, such as Autistic Symptoms Rating Scale.
Composite	A compound score of more than one outcome or instrument total scores based on the instrument employed.
Other	Any outcome that does not belong to the above-mentioned groups.

## Results

**Fig. 1:**
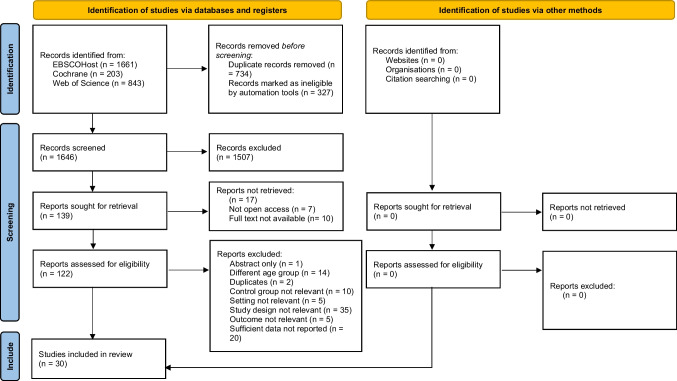
Detailed study selection process using PRISMA 2020 flow diagram

The search yielded 2,380 citations, of which 734 duplicates were excluded. The remaining 1,646 records were screened by title and abstract, resulting in 139 eligible studies. As 17 articles were not accessible, 122 were screened in full text, of which 30 were included in the data analysis (Fig. 1).

**Fig. 2:**
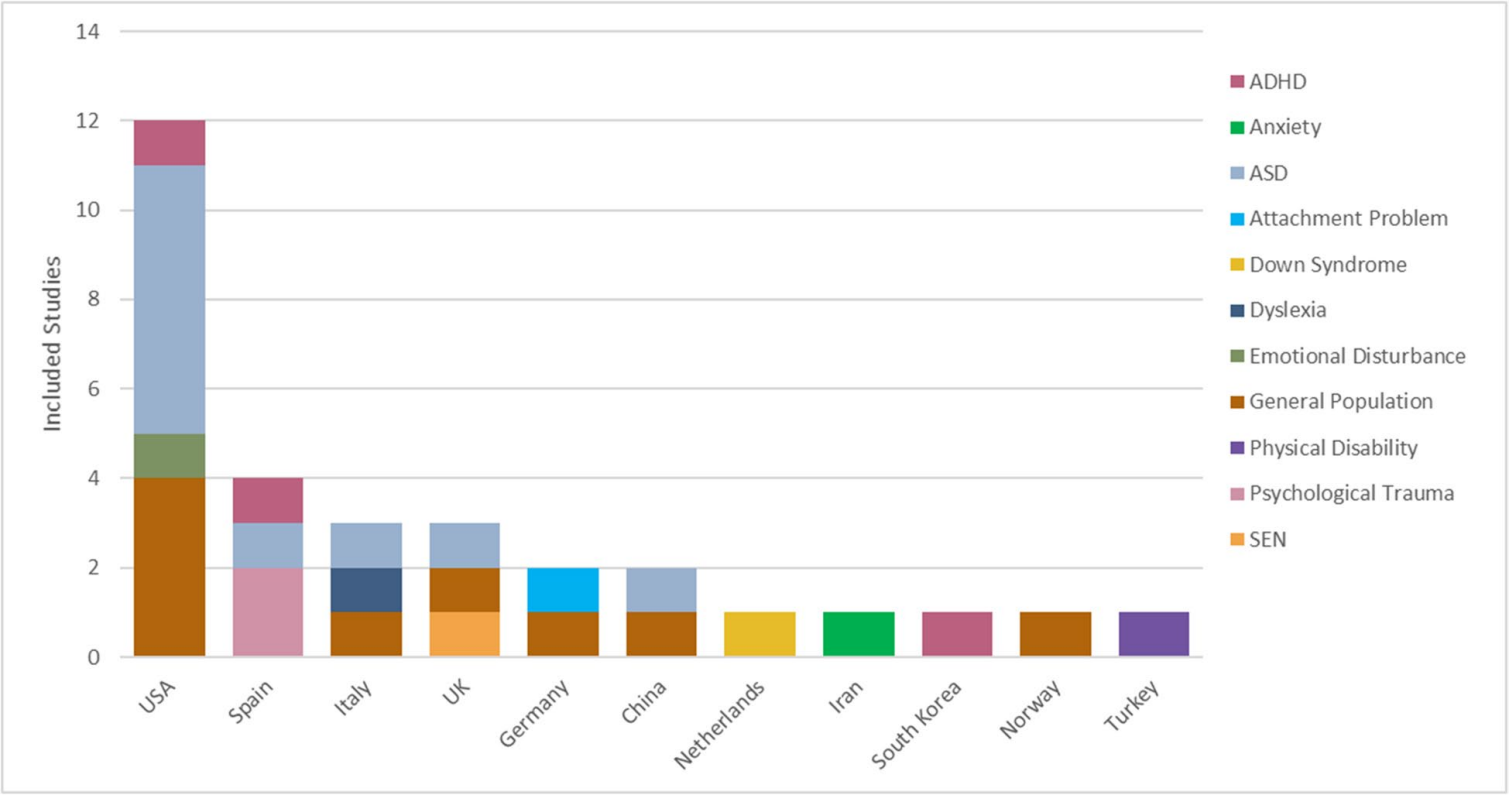
Included studies by country and study population

Table 4 groups the main characteristics of the studies by study population. Of the 30 articles, one involves two studies [41], hence resulting in 31 studies in the data extraction. Among them, 22 were RCTs, and 7 were quasi-experimental. The remaining two were a semi cross-over, and an observational study [25, 42]. Studies were principally conducted in the USA (*n* = 12), and Europe (Spain, *n* = 4; Italy, *n* = 3; Germany, *n* = 2; UK, *n* = 2; Netherlands, *n* = 1; Norway, *n* = 1), with a few in Asia (China, *n* = 2; Turkey, *n* = 1, South Korea, *n* = 1). Study populations ranged from general school-aged children (*n* = 8) to specific populations, such as children with ASD (*n* = 10) and ADHD (*n* = 3) as shown in Fig. 2.

**Table 4:** Study characteristics of included studies

Reference	Study Design	Country	Sample size^a^	Age (SD)^b^	Control	Intervention	Setting
**ASD**							
Bass et al. [43]	RCT	USA	I = 19, C = 15	4–10	Waitlist	Therapeutic horse riding and horsemanship skills 1 h per week for 12 weeks	Riding Center
Becker et al. [44]	Quasi Experimental	USA	I = 17, C = 14	10.97 (1.84)	Traditional social skills training 1 h per week × 12 sessions (active control)	Social skills training with dogs 1 h per week for 12 weeks	School
Borgi et al. [45]	RCT	Italy	I = 15, C = 14	8.6 (1.7)	Waitlist	Equine assisted therapy (horse riding and grooming activities) 1 h per week for 6 months	Riding Center
Gabriels et al. [46]	RCT	USA	I = 58, C = 58	6–16	Horsemanship skills taught with life-sized stuffed horses (active control)	Therapeutic horse riding and horsemanship skills 45 min per session for 10 weeks	Riding Center
Gabriels et al. [47]	RCT	USA	I = 36, C = 28	6–16	Horsemanship skills taught with life-sized stuffed horses (active control)	Therapeutic horse riding and horsemanship skills 45 min per session for 10 weeks	Riding Center
García-Gómez et al. [48]	Quasi Experimental	Spain	I = 8, C = 8	7–14	Conventional treatment (medical and re-education)	Therapeutic horse riding 45 min × 24 sessions; 2 per week for 3 months	Riding Center
Harris and Williams [42]	Observational	UK	I = 10, C = 14	7.38 (0.74)	Education as usual	Horse riding 45 min per week for 7 weeks	School
Pan et al. [49]	RCT	USA	I = 8, C = 8	6–16	Barn activities with stuffed horse 45 min per week (active control)	Therapeutic horse riding and non-riding activities 45 min per week for 10 weeks	Riding Center
Peters et al. [50]	RCT	USA	I = 11, C = 9	6–13	Occupational therapy in a garden environment 60 min × 10 sessions (active control)	OTEE HORSPLAY – Occupational therapy and activities with horses 60 min × 10 sessions for 10 weeks	Riding Center
Zhao et al. [51]	RCT	China	I = 31, C = 30	7.10 (1.42)	Routine therapy	Therapeutic horse riding and horsemanship skills 60 min session; 2 per week for 16 weeks	Riding Center
**General Population**							
Beetz [52]	Quasi Experimental	Germany	I = 25, C = 21	8.5 (0.51)	Education as usual	Dog presence in classroom 1 day per week for the entire school year	School
Crossman et al. [53]	RCT	USA	I = 26, C = 26	10–13	Waitlist	Interaction with dogs for 15 min in children induced with psychosocial stress	Lab Facility
Hauge et al. [54]	RCT	Norway	I = 24, C = 25	13.5 (NR)	Waitlist	Horse riding and non-riding activities 2 h session per week for 4 months	Stables
Lenihan et al. [11]	RCT	USA	I = 9, C = 6	NR	Reading to human volunteer (active control)	Reading to dogs 1 h per week for 5 weeks	School
Linder et al. [55]	RCT	USA	I = 14, C = 14	NR	Education as usual	Reading to therapy dogs after school 30 min per week for 6 weeks	School
Meints et al. [41]	RCT	UK	NR	8–9 (Study 1)	Education as usual	Dog-assisted learning intervention 20 min × 2 sessions per week for 4 weeks	School
Ngai et al. [13]	Quasi Experimental	China	I = 55, C = 55	NR	Education as usual	CARing Kids – Dog-assisted program on socio-emotional competence 75 min × 6 sessions	School
Pendry et al. [56]	RCT	USA	I = 44, C = 51	11.34 (NR)	Waitlist	Horse riding and non-riding activities 90 min per week for 11 weeks	Riding Center
Scandurra et al. [57]	Quasi Experimental	Italy	I = 63, C = 41	6.55 (0.50)	Education as usual	Dog-assisted education 1 h × 5 group sessions; bimonthly sessions for 3 months	School
**ADHD**							
García-Gómez et al. [58]	RCT	Spain	I = 9, C = 5	7–14	Education as usual	Therapeutic horse riding 45 min × 24 sessions; 2 sessions per week for 3 months	Riding Center
Oh et al. [59]	RCT	South Korea	I = 17, C = 15	6–12	Medication using methylphenidate or atomoxetine	Horse riding and horsemanship skills 1 h × 24 sessions; 2 sessions per week for 3 months	Riding Center
Schuck et al. [24]	RCT	USA	I = 12, C = 12	7–9	Cognitive-behavioral group therapy without dog (active control)	Dog-assisted intervention with cognitive-behavioral group therapy 4 h 30 min per week for 3 months	School
**Special Education Needs (SEN)**							
Meints et al. [41]	RCT	UK	NR	8–11 (Study 2)	Education as usual	Dog-assisted learning intervention 20 min × 2 sessions per week for 4 weeks	School
**Psychological Trauma**							
Balluerka et al. [60]	Quasi Experimental	Spain	I = 39, C = 24	15.27 (1.63)	Usual residential care program	Dog- and horse-assisted psychotherapy 2 consecutive days × 34 sessions; 1 session per week for 12 weeks	Farm
Muela et al. [61]	Quasi Experimental	Spain	I = 52, C = 35	12–17	Usual residential care program	Dog- and horse-assisted psychotherapy 2 consecutive days × 34 sessions; 1 session per week for 12 weeks	Farm
**Attachment Problems**							
Beetz et al. [62]	RCT	Germany	I = 24, C = 10	7–11	Human as social supporters in children induced with psychosocial stress (active control)	1 h session of dogs as social supporters in children induced with psychosocial stress	School
**Dyslexia**							
Corallo et al. [63]	RCT	Italy	I = 8, C = 8	7–12	Traditional neuropsychological training (active control)	Neuropsychological training with donkey assisted therapy; 1 session per week for 6 months	NR
**Physical Disabilities**							
Demiralay and Keser [64]	RCT	Turkey	I = 21, C = 23	8–11	Routine education in rehab center	Individual therapeutic sessions with cats 45–60 min per week for 7 weeks	Rehabilitation centers
**Down Syndrome**							
Griffioen and Enders-Slegers [25]	Semi Crossover	Netherlands	I = 18, C = 10	6–11	Therapy in swimming pool without dolphin; 1 h per week for 6 weeks (active control)	Dolphin-assisted therapy 1 h per week for 6 weeks	Dolphin Aquarium
**Emotional Disturbance**							
Murry and Allen [65]	RCT	US	I = 16, C = 19	12 (NR)	Support meeting with therapist (active control)	Support group meetings with therapist and reptiles; 75 min per week for 16 weeks	Residential Care
**Anxiety**							
Tahan et al. [66]	RCT	Iran	I = 10, C = 10	5–7	No treatment	Animal-assisted therapy 90 min × 8 sessions in 8 days	NR

^a^Sample sizes are noted separately for intervention (I) and control (C)

^b^Ranges are provided when age means and standard deviations are not reported (NR)

**Fig. 3:**
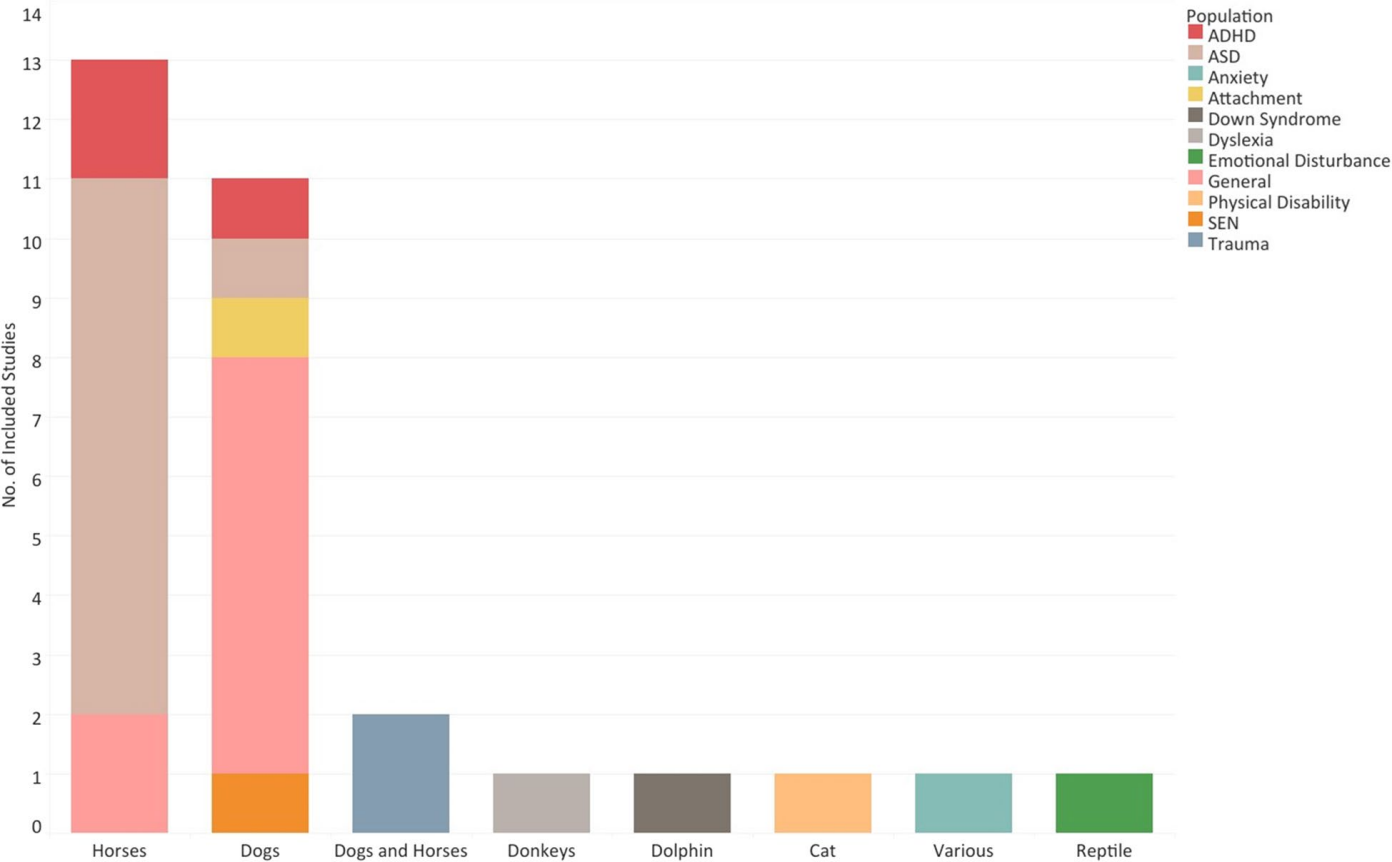
Animals employed in the included studies by study population

Many interventions were implemented in riding centers or stables (*n* = 12) and schools (*n* = 11), with a minority in farms (*n* = 2) and other settings (*n* = 4). Horses (*n* = 13) and dogs (*n* = 11) were the most used animals (Fig. 3). In terms of children with ASD, horses were the most commonly employed, while only one study used dogs [44]. Similarly, studies involving children with ADHD used horses, while Schuck et al. [24] included dogs in group cognitive behavioral therapy sessions [24]. Most studies in the general population employed dogs, while the remaining two studies used horses [54, 56]. In other populations, such as children with Down syndrome and emotional disturbance, dolphins [25] and reptiles [65] were used. Both horses and dogs were used for children with psychological trauma [60, 61].

Interventions typically involved multiple sessions lasting from 8 days to an academic year [52, 66]. Two studies examined immediate effects on cortisol level by inducing psychosocial stress (through administering separation anxiety test, and tier social stress test) in children to measure the impact of dog interactions [53, 62]. In ADHD populations, the intervention duration was consistent at 3 months [24, 58, 59], while ASD population varied from 7 weeks to 6 months [42, 45]. The majority of included studies employed a passive control (*n* = 19), such as usual education, waitlist, or no treatment. Eleven studies used active controls, with some substituting the animal with stuffed animals [46, 47, 49], while others used human stand-ins for the control group [11, 62].

Outcomes measured varied widely across studies as shown in Table 5. ADHD and ASD studies mainly measured abilities, externalizing, and internalizing behaviors, with common behavioral outcomes like hyperactivity, irritability, somatic complaints, withdrawal, social skills, attention problems, and communication. Learning abilities and attention problems were assessed in children with dyslexia, trauma, and general population [11, 55, 56, 60, 61, 63, 65]. Concerning attitudes, studies reported attitudes towards the environment, school, self, or academic and recreational reading [11, 52–55, 59–62]. Well-being was measured as quality of life [59] or cortisol stress level [41, 49, 50, 53, 62, 64]. Measuring instruments overlap was limited due to diverse outcomes and populations. ASD studies frequently used the Aberrant Behavior Checklist (ABC; *n* = 5) [42, 46, 47, 49, 50], while ADHD population employed varying instruments, such as Child Behavior Checklist (CBCL), Self-Esteem Scale (SES), Behavior Assessment System for Children (BASC), Social Competence Inventory (SCI), and Social Skills Improvement System Rating Scales (SSIS-RS) [24, 58, 59]. General population studies often used the Elementary Reading Attitude Survey [11, 55]. Additionally, the studies used various outcome assessors. Most outcome measures for children with ASD were reported by caregivers or teachers, utilizing tools such as the ABC checklist, BASC, VABS, and SRS. Similarly, in the ADHD population, the commonly used measures including the CBCL, BASC, and SSIS-RS were primarily reported by caregivers or teachers. In contrast, self-reported measures were more prevalent in studies involving the general population (Table 5).

**Table 5:**
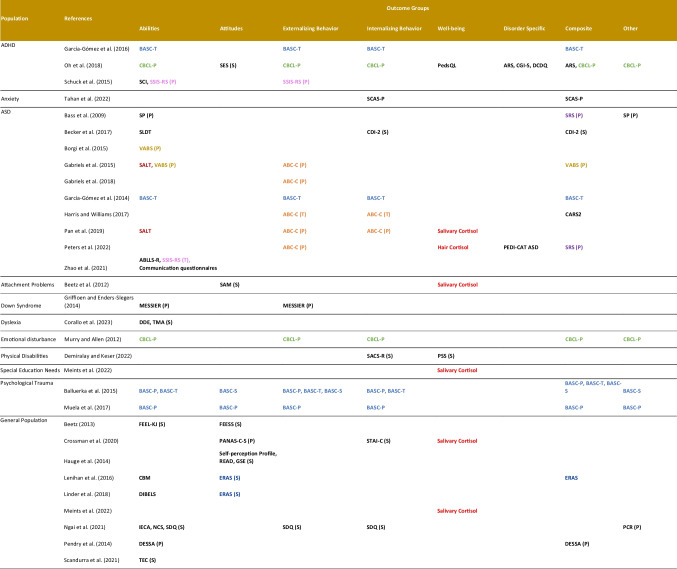
Instruments employed and outcomes groups measured in each study by population with information if instruments were self-reported (S), parent-reported (P) or teacher-reported (T)

Abbreviations: ABC-C: Aberrant Behavior Checklist-Community; ABLLS-R: Assessment of Basic Language and Learning Skills-Revised; ARS: ADHD Rating Scale; ASD: Autism Spectrum Disorder; ADHD: Attention-Deficit/Hyperactivity Disorder; BASC-P: Behavior Assessment System for Children (Parent-reported); BASC-T: Behavior Assessment System for Children (Teacher-reported); BASC-S: Behavior Assessment System for Children (Self-reported); CARS2: Childhood Autism Rating Scale, Second Edition; CBCL-P: Child Behavior Checklist-Parent version; CBM: Curriculum-Based Measurement; CDI-2: Children’s Depression Inventory Second Edition; CGI-S: Clinical Global Impressions Severity; DCDQ: Developmental Coordination Disorder Questionnaire; DDE: Evaluation of Dyslexia and Dysorthography; DESSA: Devereux Student Strength Assessment; DIBELS: Dynamic Indicators of Basic Early Literacy Skills; ERAS: Elementary Reading Attitude Survey; FEEL-KJ: Questionnaire on emotion regulation in children and juveniles; FEESS: Questionnaire on emotional and social experiences in school, grade 3–4; GSE: General Self-Efficacy scale for adolescents; Self-Perception Profile: Susan Harter’s self-perception profile for adolescents to measure self-esteem; IECA: Index of Empathy for Children and Adolescents; MESSIER: Matson Evaluation of Social Skills for Individuals with Severe Retardation; NCS: Need for Cognition Scale; PEDI-CAT ASD: Pediatric Evaluation of Disability Inventory Computer Adaptive Test, Autism Spectrum Disorder Module; PedsQL: Pediatric Quality of Life Inventory; PCR: Parent–Child Relationship Scale; PSS: Perceived Stress Scale; PANAS-C-S: Positive and Negative Affect Schedule for Children, Short Form; READ: Resilience Scale for Adolescents; SAM: Self-Assessment Manikin; SACS-R: Social Anxiety in Children Scale-Revised Version; SALT: Systematic Analysis of Language Transcripts; SCAS-P: Spence Children Anxiety Scale for parents; SCI: Social Competence Inventory; SDQ: Strength and Difficulty Questionnaire; SES: Self-Esteem Scale; SLDT: Social Language Development Test; SP: Sensory Profile; SRS: Social Responsiveness Scale; SSIS-RS: Social Skills Improvement System–Rating Scales; STAI-C: State/Trait Anxiety Inventory for Children; TEC: Test of Emotion Comprehension; TMA: Multidimensional Self-Esteem Test; VABS: Vineland Adaptive Behavioral Scale

Twenty-five out of 30 studies reported significant positive effects in the AAS group compared to controls, as summarized in Table 6.. For ASD populations, AAS groups showed improvements in abilities, externalizing, and internalizing behaviors, such as reduced hyperactivity, irritability, and anxiety, and improved social skills, motivation, and self-control [42–51]. In the general population, AAS led to better attitudes toward school, positive emotions, cognitive competence, empathy, social competence, and self-awareness [13, 52–54, 56, 57]. Among ADHD populations, only Oh et al. [59] reported significant decrease in thought problems, such as obsessive thinking, hearing or seeing things, repetitive acts, and strange behaviors, in the horse-riding group compared to pharmacotherapy [59], while others found no significant group differences but noted time-dependent improvements in behavior and social skills [24, 58].

**Table 6.**
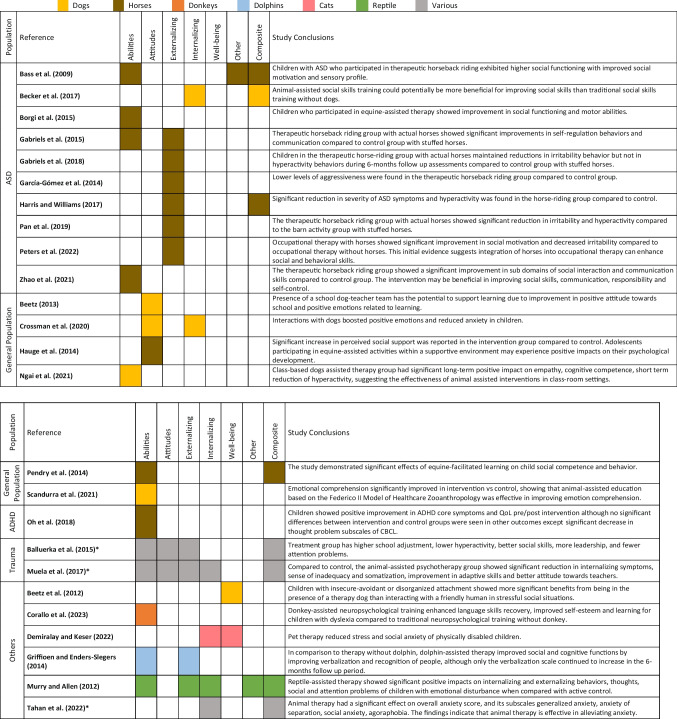
Main results from studies that showed effects on at least one outcome of interest

*Tahan et al. [66] employed various animals; Muela et al. [61] and Balluerka et al. [60] employed both horses and dogs

Abbreviations: ADHD: Attention-Deficit/Hyperactivity Disorder; ASD: Autism Spectrum Disorder; CBCL: Child Behavior Checklist; QoL: Quality of life

Children with psychological trauma showed better school adjustment, social skills, leadership, and reduced hyperactivity and attention problems [60, 61]. Positive effects on behaviors and social skills were also observed in children with emotional disturbances [65]. Griffioen and Enders-Slegers [25] also reported significant improvements in abilities and externalizing behaviors in children with Down syndrome through dolphin-assisted therapy [25]. Corallo et al. [63] observed increased learning and self-esteem in children participating in donkey-assisted therapy compared to traditional neuropsychological groups [63]. Beetz et al. [62] found significantly lower cortisol levels in children with attachment problems using dogs as social supporters compared to human-supporters [62]. Tahan et al. [66] and Demiralay and Keser [64] reported significant reductions in anxiety in those who underwent AAS compared with the controls [64, 66].Detailed results of each included study can be found in the supplementary Online Resource.

**Fig. 4:**
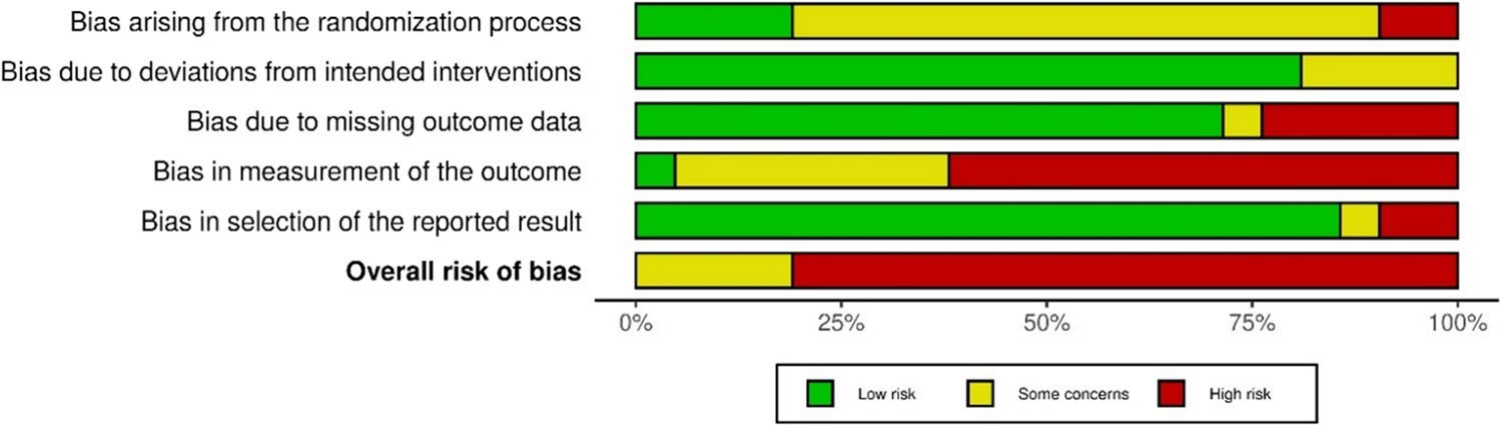
Summary plot showing risk of bias of randomized controlled trials

Among RCTs, 81% (*n* = 17) were found to have high risk of bias overall (Fig. 4). High bias was noted in non-blinded outcome assessors (*n* = 13) and inadequately addressed missing data (*n* = 5). Many studies did not report on allocation concealment, resulting in some concerns of bias associated with randomization process. However, low risk of bias was seen in the domain deviations from intended interventions and selective reporting.

**Fig. 5:**
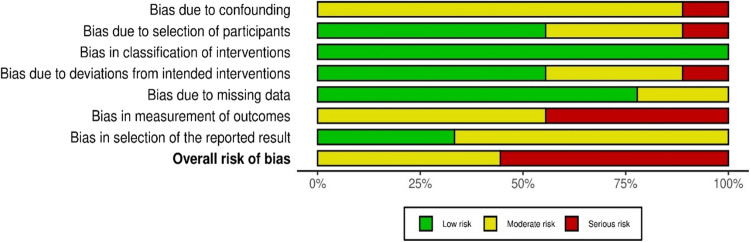
Summary plot depicting risk of bias of non-randomized controlled trials

For non-RCTs (*n* = 9), five studies had high overall risk, and four had moderate risk (Fig. 5). Most had moderate or serious bias in outcome measurement due to non-blinded assessors. Likewise, moderate risk was seen in baseline confounding in most studies (*n* = 8). Low risk was found in intervention classification and missing data, indicating clearly defined groups and well-handled missing data.

## Discussion

The current systematic review provides updated results on the effects of AAS on the well-being and socio-emotional functioning of school-aged children. A major challenge in interpreting the findings is the high heterogeneity across study populations, interventions, outcomes, and measurement instruments, a common issue in similar research [27–29], and pose a problem when trying to draw conclusions about the effectiveness of AAS. Even in studies on similar populations, such as children with ASD, authors employed a wide array of instruments, including Sensory Profile (SP), Vineland Adaptive Behavioral Scales (VABS-II), and Systematic Analysis of Language Transcripts (SALT). While this review highlights improvements in motivation, engagement, and social skills, potentially enhancing school attendance and academic performance in children, these findings should be interpreted with caution due to methodological limitations and the high risk of bias present in the included studies.

Similar to previous reviews, therapeutic horse riding is the most common intervention in children with ASD [18, 67]. Improvements were seen in abilities, externalizing and internalizing behaviors; children displayed better social and communication skills, motor skills, sensory profiles, and self-regulation in addition to reduced irritability and hyperactivity, compared to children who did not partake in AAS. However, assessments relied mainly on caregivers and teachers who were unblinded to the intervention, which introduces subjectivity and could inflate the perceived effectiveness of AAS (Table 5).

In the general population, studies employed instruments that measured social and emotional competence such as Devereux Student Strength Assessment (DESSA) as well as instruments that focused on literacy, namely Dynamic Indicators of Basic Early Literacy Skill (DIBELS) and Curriculum Based Measurement (CBM). Overall, children involved in AAS showed potential improvements in abilities, attitudes towards school and learning, cognitive competence, emotion comprehension, and reduced anxiety. This finding is similar to Brelsford et al. [16] review of animal-assisted intervention in educational settings, which reported positive effects on cognitive and socio-emotional behavior [16]. However, not all studies recorded significant results [11, 41, 55].

Evidence for the effectiveness of AAS in other populations was more limited. In children with ADHD, only one study found significant improvement in thought problems within the AAS group when compared to pharmacotherapy [59]. However, nonblinded assessors were involved. Conversely, a study with blinded assessors that found positive effects was excluded from this review due to the absence of an appropriate control [68].

It is noteworthy to discuss the various control groups used across the included studies. Passive control groups, where children either received no treatment, standard treatment or were placed in a waitlist, were more frequent than active control groups, where children engaged in similar activities as the interventions but without the animal. López-Cepero [69] argued that comparing AAS to passive controls allows assessment of the overall intervention impact but fails to distinguish between specific contributions of each intervention component [69]. This approach further faces criticism in the AAS field as passive controls do not control for non-specific treatment effects, compromising internal validity [69, 70]. To address this, Kazdin [71] and López-Cepero [69] advocated for active control groups to mitigate participant biases and control non-specific effects [69, 71]. For example, control groups could engage in similar activities as the experiment groups but without animals, or AAS could be integrated as a supplementary component to existing evidence-based interventions [69]. Such designs are critical for isolating the unique contributions of human-animal interaction.

In the current review, nine studies employed active control groups and reported positive outcomes specifically attributable to human-animal interactions, highlighting the potential efficacy of AAS when compared to similar non-animal-based interventions. These findings suggest that the observed effects in studies using active controls may better reflect the specific contributions of animals in AAS, as opposed to the passive controls. (Table 4, Table 6.) [25, 44, 46, 47, 49, 50, 62, 63, 65].

There is a growing trend towards more rigorous research in AAS, with most studies being randomized controlled trials (RCTs). Increased use of RCTs can reduce bias and improve the validity of findings when conducted carefully. However, several RCTs had small sample sizes (fewer than 12 children per group), which can affect study power and quality [11, 24, 31, 49, 50, 58, 63, 66]. The risk of bias assessment of the included studies showed blinding of outcome assessors being a common concern. This issue was noted in 8 RCTs and 2 non-RCTs with a high risk of bias. Although blinding is often challenging in studies involving children—where assessments frequently rely on reports from parents, teachers, or caregivers—approaches such as using independent or masked assessors could help limit bias in future research. This is particularly important to reduce potential influences of subjective expectations on reported outcomes [72, 73]. Moreover, 4 RCTs and 4 non-RCTs were found to have moderate risk of bias, primarily due to issues in the randomization process and outcome measurement domain [11, 13, 44, 53–55, 60, 61].

While the methodological limitations of the studies included in this review warrant cautious interpretation, there is some evidence suggesting that AAS may have potential benefits for various behavioral and socio-emotional aspects of school-aged children. These findings are consistent with the theoretical framework proposed by Gee et al. [17], which suggests that incorporating animals in interventions enhances learning by improving self-efficacy, attention, social interaction, and reducing anxiety [15]. However, further research with higher methodological rigor is needed to confirm these effects.

In addition to quality, transferability and the ability to draw conclusions from studies conducted in different countries require careful assessment [74, 75]. AAS encompassing several areas, coupled with different implementation settings, various measurement tools, and diverse study populations in our review add to this challenge. However, reporting remains a critical issue for transferability of AAS, as sufficient descriptions are needed for comparisons [76]. None of the studies in our review reported or discussed the costs associated with such interventions, which subsequently hinders the implementation and transferability to real world settings, as resources and costs are typically a principal consideration. The need of standardized generic measurement tools, cost transparency and economic analyses to better inform decision-making have been highlighted by previous studies [26, 29, 69].

Most studies were conducted in high-income settings. This prevalence can be explained by factors such as more resources, trained professionals, research funding, and social acceptance of animals in therapeutic settings [77–79]. While structural systems can be detrimental with regards to transferability, they arguably play a more subordinate role as AAS regulations continue to develop [80, 81]. However, the extent at which contextual reasons impact transferability may vary widely.

Lastly, animal welfare is a significant concern, as some studies have involved dolphins and reptiles, which IAHAIO (International Association of Human-Animal Interaction Organizations) advises against in favor of using domesticated animals [25, 65, 82, 83]. Similar considerations have been raised by various authors, emphasizing the need for familiar environments and strong handler relationships to ensure animal well-being [83, 84]. Despite this growing emphasis on animal welfare, nearly half of the studies did not mention ethical considerations at all, indicating it is often overlooked in AAS design. Continuous monitoring of animal welfare, standard training protocols of animals to be used in interventions, and development of techniques to assess fatigue and stress are crucial to ensure optimal outcomes for both animals and humans.

This review possesses several strengths. It includes only peer-reviewed studies with control groups, most of which are RCTs or quasi-experimental designs. The review was registered in PROSPERO and adhered to the PRISMA guidelines for clear reporting. A comprehensive search was employed across multiple databases. However, the review also has limitations. Half of the studies had small sample sizes, limiting generalizability. Most studies were conducted in high-income countries, reducing applicability to low- and middle-income settings. Moreover, heterogeneity across outcomes and studies did not allow for a meta-analysis, which limits the ability to draw definitive conclusions.

## Conclusion

While there is evidence supporting the benefits of animal-assisted services (AAS) for improving the socio-emotional and behavioral well-being of school-aged children, the inconsistencies and potential bias across the studies reviewed demand caution in interpreting these effects. Future research must address key study design issues to reduce bias and more clearly establish the efficacy of AAS. Another consideration is the costs and cost-effectiveness of implementing AAS. Conducting health economic analyses to examine the costs and benefits of AAS will be crucial next steps to provide more concrete evidence for decision-makers. Lastly, ensuring the welfare of the animals involved will be essential to maintaining ethical integrity in AAS.

## Supplementary Information

Below is the link to the electronic supplementary material.

Supplementary file1 (DOCX 1635 KB)

## Data Availability

No datasets were generated or analysed during the current study.

## Declarations

### Data sharing

The corresponding author can be contacted for any data not included in the article or supplementary material. Data will be provided based on the nature of the research request.
