# Supplementary material for: Effectiveness of animal-assisted services for school-aged children: a systematic review
Source: Eur Child Adolesc Psychiatry. 2025 Jun 2;34(10):3017–33. doi: 10.1007/s00787-025-02740-7 (PMC12592290; doi:10.1007/s00787-025-02740-7)

Effectiveness of Animal-Assisted Services for School-Aged Children: A Systematic Review (Supplementary Information)

**Journal:** European Child and Adolescent Psychiatry

**Authors**: Ingyin Moe^1^, Pei Ju Ho^1^, Maria Andersson^2^, Sara Karlberg^2^, Lena Lidfors^2^, Filipa Sampaio^1^, Inna Feldman^1^

**Affiliations**

^1^ Department of Public Health and Caring Sciences, Uppsala University, Uppsala, Sweden

^2^ Department of Applied Animal Science and Welfare, Swedish University of Agricultural Sciences, Sweden

**Corresponding author**

Dr Ingyin Moe

Department of Public Health and Caring Sciences

Uppsala University

Husargatan 3, 751 22 Uppsala, Sweden

[ingyin.moe@uu.se](mailto:ingyin.moe@uu.se)

+46(0)765669724

Table of Contents

[**Search Strategy** 3](#_Toc180521654)

[**Table 1. Selection Criteria** 3](#_Toc180521655)

[**Figure 1. Detailed main results of included studies** 4](#_Toc180521656)

## **Search Strategy**

The search was performed in the following electronic databases from their inception date through August 2023: PubMed/Medline (1966—Present), PsycINFO (1840—Present), PsycARTICLES (1894— Present), CINAHL (1961—Present) through EBSCOHost, Cochrane Database of Systematic Reviews (1996—Present), Web of Science (1964—Present).

**Term 1 AND Term 2 AND Term 3**

**Term 1:** **child* or kid* or “school age*” or youth or adolescen* or “young people”**

**Term 2:** **“animal assisted” or “dog assisted” or “equine assisted” or “pet assisted” or “canine assisted” or “hippotherap*” or “pet therap*” or “animal therap*” or "assistan* dog*" or "horseback riding" or "pet facilitated" or "therapeutic animal*" or "therapeutic horse*" or "therapy with animal*" or "dolphin assisted"**

**Term 3:** **"school performance" or "performance" or "school achievement*" or "school outcome*" or "academic achievement*" or "academic performance" or "learning" or "behavio* change" or "behavio*" or "school attend*" or “presen*” or “absent*” or “grade*” or “literac*” or “social skill*” or "quality of life" or “read*” or “class attend*” or “school absen*” or “class performance” or “cognitive skill*” or “motivation” or “anxiet*” or “student engag*” or “mental health” or “ well being”**

## **Table 1. Selection Criteria**

| **Inclusion Criteria** | **Exclusion Criteria** |
| --- | --- |
| - Articles in English, Portuguese, Spanish,  Italian, French, Chinese, and Russian  - Peer-reviewed articles  - School-aged children that are 5-18 years old  - Any animal-assisted intervention  - Studies with interventions conducted either at school or outside of school, and measuring school-related outcomes  - RCTs or observational studies with a control group and pre-and follow-up measurements | - Articles that are not in English, Portuguese,  Spanish, Italian, French, Chinese, and Russian  - Articles that are not peer-reviewed  - Study populations who are not school-age and  between 5-18 years old  - Case studies, case reports, and grey literature  - Studies that are conducted in hospitals or  health care settings  - Interventions with robotic animals  - Studies without a control group |

## **Figure 1. Detailed main results of included studies**


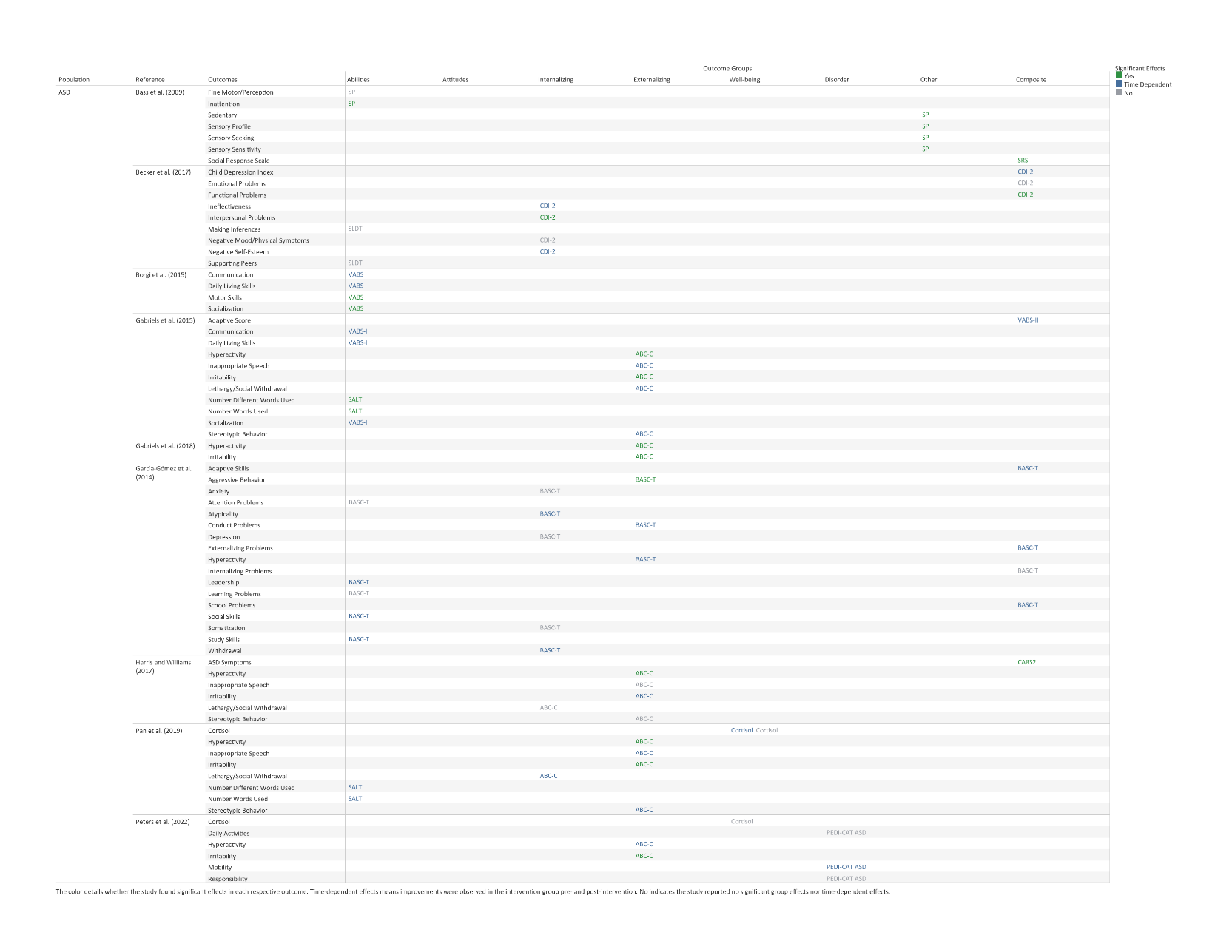

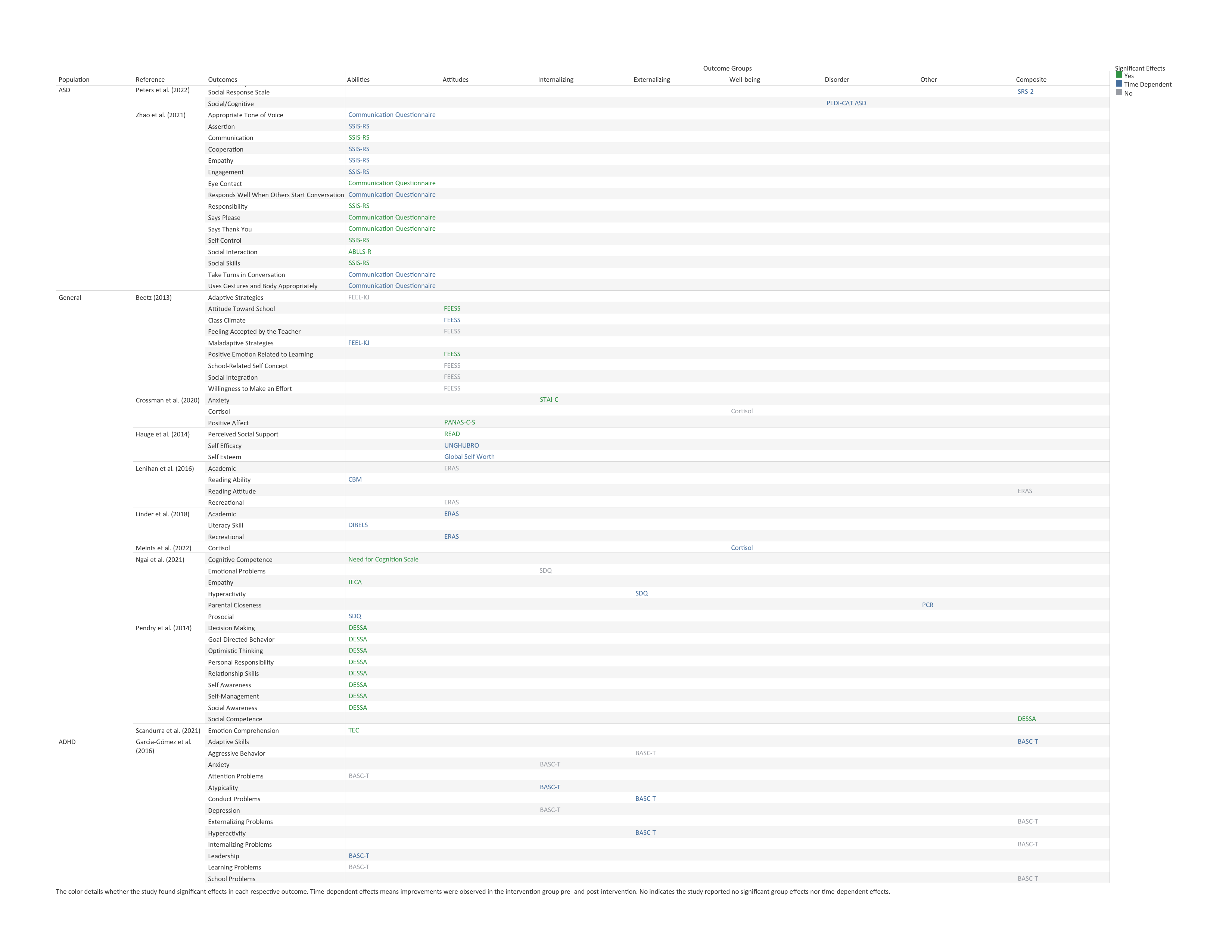

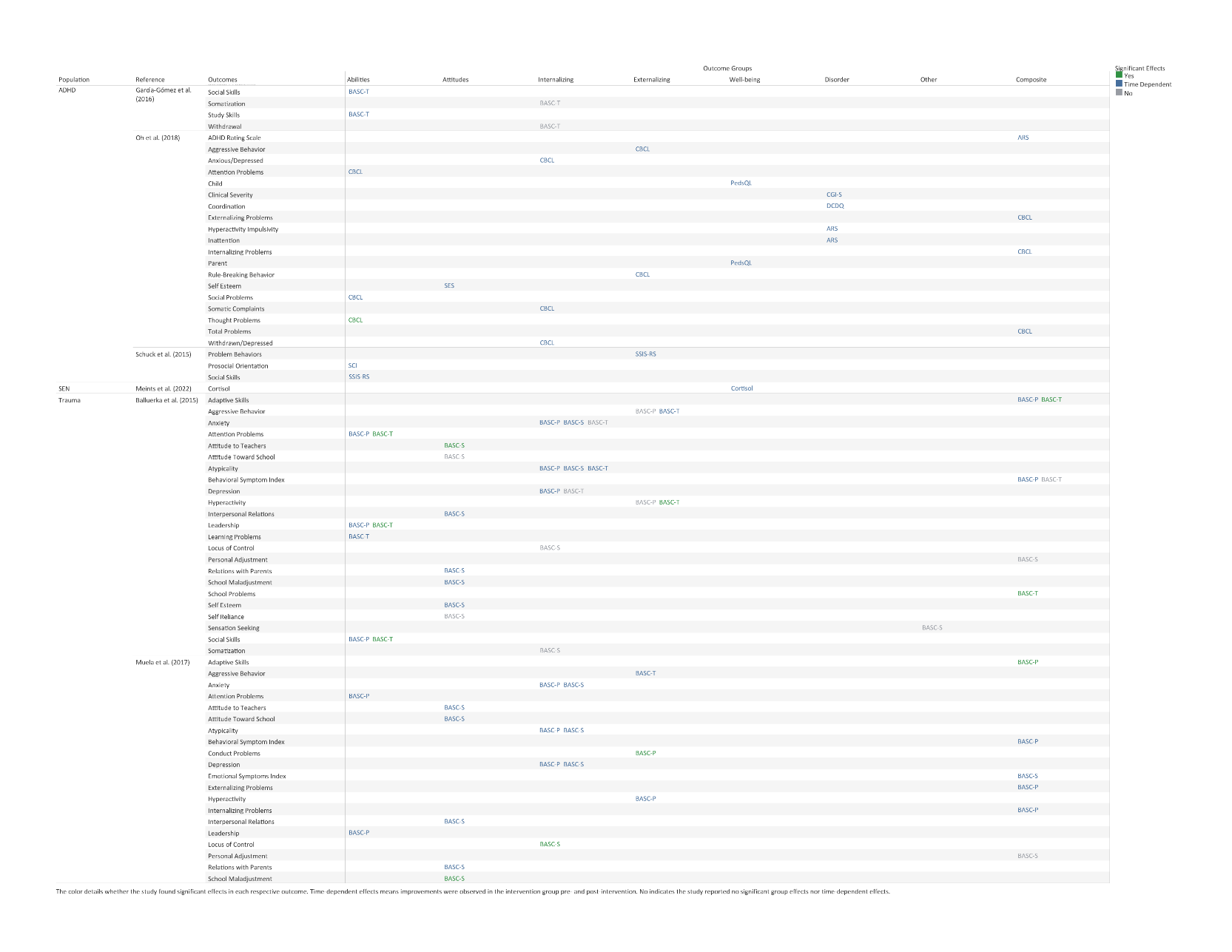

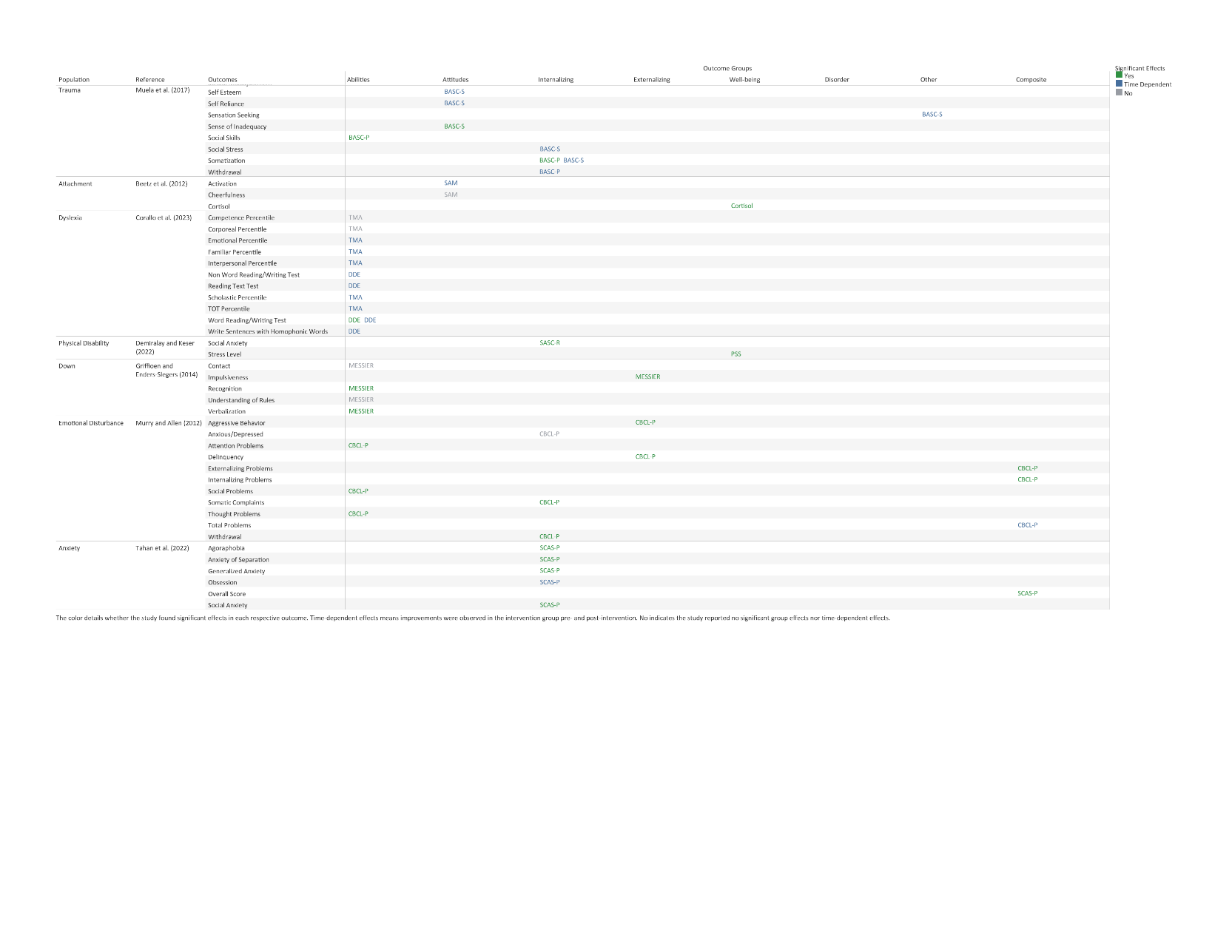

Supplement: Supplementary file 1 — Supplementary file1 (DOCX 1635 KB) [file 787_2025_2740_MOESM1_ESM.docx]
